# Vegetable Butters and Oils as Therapeutically and Cosmetically Active Ingredients for Dermal Use: A Review of Clinical Studies

**DOI:** 10.3389/fphar.2022.868461

**Published:** 2022-04-25

**Authors:** Nina Poljšak, Nina Kočevar Glavač

**Affiliations:** Department of Pharmaceutical Biology, Faculty of Pharmacy, University of Ljubljana, Ljubljana, Slovenia

**Keywords:** vegetable butters and oils, skin barrier, fatty acids, infant skin, xerosis, atopic dermatitis, psoriasis

## Abstract

While the chemical composition of vegetable butters and oils has been studied in detail, there is limited knowledge about their mechanisms of action after application on the skin. To understand their dermal effects better, 27 clinical studies evaluating 17 vegetable oils (almond, argan, avocado, borage, coconut, evening primrose, kukui, marula, mustard, neem, olive, rapeseed, sacha inchi, safflower, shea butter, soybean and sunflower oils) were reviewed in this research. The reviewed studies focused on non-affected skin, infant skin, psoriasis, xerosis, UVB-induced erythema, atopic dermatitis, *molluscum contagiosum*, tungiasis, scars, striae and striae gravidarum. We conclude that in inflammation-affected skin, vegetable oils with a high content of oleic acid, together with the lack of or a low linoleic acid content, may cause additional structural damage of the stratum corneum, while oils high in linoleic acid and saturated fatty acids may express positive effects. Non-affected skin, in contrast, may not react negatively to oils high in oleic acid. However, the frequency and duration of an oil’s use must be considered an important factor that may accelerate or enhance the negative effects on the skin’s structural integrity.

## Introduction

Vegetable butters and oils have been used for centuries for their positive therapeutic and cosmetic effects on the skin’s health, and are also extensively used in the pharmaceutical and cosmetic industries today. They function, for example, as active ingredients, excipients and extraction solvents.

**FIGURE 1:**
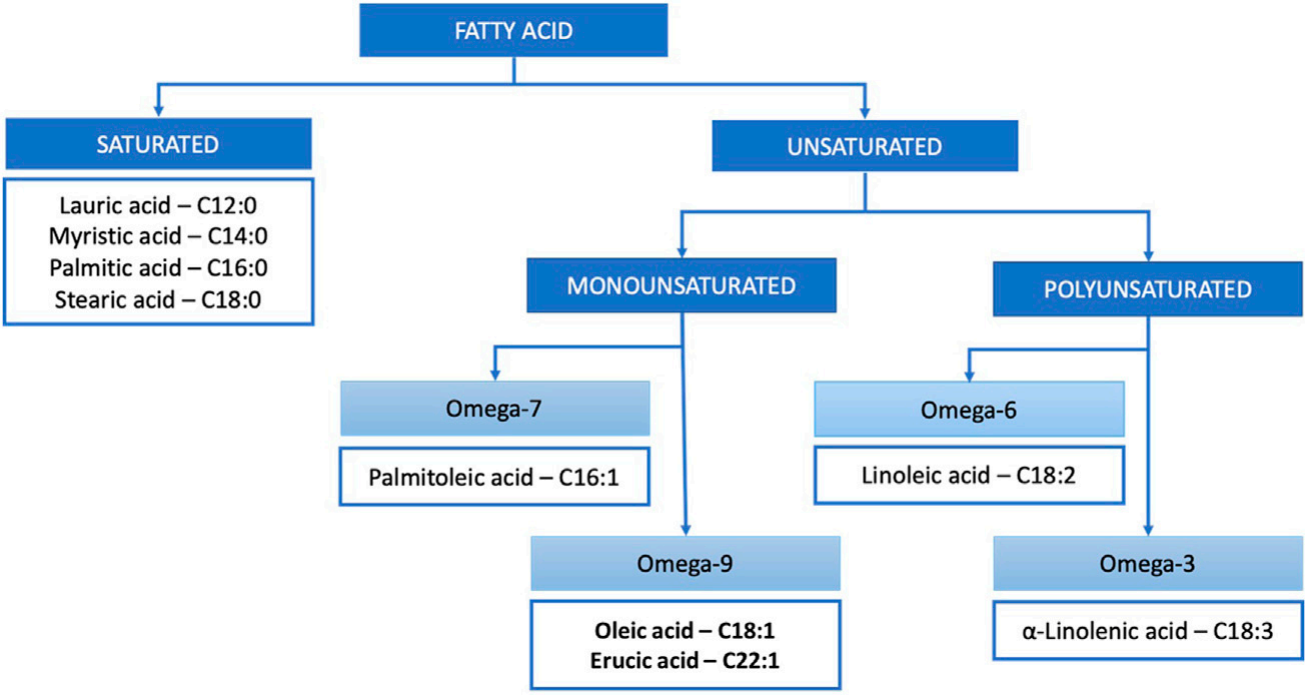
Classification of the most common fatty acids occurring in vegetable butters and oils.

In terms of chemistry, vegetable butters and oils are composed of triglycerides (typically around 99%) and unsaponifiable matter (typically around 1%). Triglycerides are ester derivatives of glycerol and fatty acids. Depending on the number of double bonds, fatty acids are classified into saturated and mono- and polyunsaturated (Figure 1), which defines their susceptibility to light-, heat- or oxygen-induced changes. The main unsaponifiable compounds are phytosterols, phenols, squalene, carotenoids and vitamin E (Janeš and Kočevar Glavač, 2018). In terms of native, complex composition, vegetable butters and oils of the highest quality are obtained through cold pressing and CO_2_ extraction, without subsequent refining, as they are not exposed to temperature- or oxidation-dependant changes, and solvent residuals are not present (Sookwong and Mahatheeranont, 2017).

The dermal effects of vegetable butters and oils are based on triglycerides and fatty acids, and unsaponifiable matter. Scientific evidence regarding the exact mechanisms of action and the extent of dermal effects is still limited. However, important progress has been made in recent years in the area of clinical research, and a growing body of evidence indicates rationale for the science-based use of vegetable butters and oils in fields such as medicine, pharmacy and cosmetic science.

This article represents the most recent review of clinical studies evaluating the use of vegetable butters and oils in the treatment and care of different skin conditions and disorders after dermal application.

## Methodology

A systematic search was performed on literature published until 2021 with PubMed, Science Direct and Google Scholar search engines. Key search words included “vegetable butter/oil”, “plant butter/oil”, “clinical study/trial”, “dermal” and “skin”. Only clinical studies evaluating the dermal effects of vegetable butters and oils were included, which resulted in 27 clinical studies. All other studies, such as studies with cosmetic or therapeutic products containing vegetable butters or oils, or studies with compounds isolated from vegetable butters or oils, were not included. Studies such as *in vitro* or *in vivo* studies not defined as clinical studies were also not included.

## Composition

The general composition of vegetable butters and oils reviewed in this article is presented in Table 1. The content of fatty acids may vary in the range of 5–10%, mainly due to the different geographical origins of plant material (Janeš and Kočevar Glavač, 2018). An even higher variability is found for unsaponifiable matter, which is typically more affected by the method of production (Poljšak and Kočevar Glavač, 2021). Selected fatty acids and their dermal effects are summarized in Table 2.

**TABLE 1:** Vegetable butters and oils, their fatty acid composition and unsaponifiable matter content; individual fatty acids were only included in the table when their content was at least 10% in at least one of the listed oils. Fatty acids of triglycerides and total unsaponifiable matter are given in percentages, “-” typically not present*.

Chain length: number of unsaturated bonds	Lauric acid (%)	Myristic acid (%)	Palmitic acid (%)	Stearic acid (%)	Palmitoleic acid (%)	Oleic acid (%)	Erucic acid (%)	Linoleic acid (%)	α-Linolenic acid (%)	Saturated fatty acids (SFA) (%) (Janeš and Kočevar Glavač, 2018)	Monounsaturated fatty acids (MUFA) (%) (Janeš and Kočevar Glavač, 2018)	Polyunsaturated fatty acids (PUFA) (%) (Janeš and Kočevar Glavač, 2018)	Unsaponifiable matter (%) (Fontanel, 2013; Janeš and Kočevar Glavač, 2018)
	C12:0	C14:0	C16:0	C18:0	C16:1, ω−7, cis	C18:1, ω−9, cis	C 22:1, ω-9, cis	C18:2, ω−6, cis	C18:3, ω−3, cis				
Coconut (*Cocos nucifera*) oil (Chowdhury et al., 1970; Fontanel, 2013; Janeš and Kočevar Glavač, 2018)	48–52	18–19	8–9	2–3	—	5–6	-	1–2	—	78–93	5–6	1–2	0.02–1.5
Almond (*Prunus dulcis*) oil (Fontanel, 2013; Mericli et al., 2017; Čolić et al., 2017; Janeš and Kočevar Glavač, 2018)	—	—	3–7.4	0.2–2.2	0.3–0.6	53–78	—	13–26	<0.7	5	75	20	0.5–1
Olive (*Olea europaea*) oil (Gunstone, 1996; Berasategi et al., 2012; Fontanel, 2013; Janeš and Kočevar Glavač, 2018)	—	—	10–12	2	1	73–78	—	7–9	1	14	74	9	0.6–3
Marula (*Sclerocarya* birrea) oil (Mariod et al., 2005; Liu and Huang, 2012; Mariod and Abdelwahab, 20121; Komane et al., 2015)	0.3	<0.3	13–15	1–9	<0.2	65–78	—	4–9	<0.7	22–26	67–70	6	0.7–3
Avocado (*Persea americana*) oil (de Oliveira et al., 2013; Fontanel, 2013; Janeš and Kočevar Glavač, 2018)	—	—	16–24	—	7	47–60	—	13–14	1	16	67	15	0.4–12.2
Canola (*Brassica napus/campestris*) oil (Gunstone, 1996; Fontanel, 2013; Lewinska et al., 2015; Janeš and Kočevar Glavač, 2018)	—	—	4	1–2	—	46–63	15	19–26	10	5	65	29	0.5–5
MUSTARD (*Sinapis alba*) OIL (Al-Jasass and Al-Jasser, 2012; Mitrović et al., 2020)	—	—	0.8–5.2	0.3–1.6	<0.2	9–25	28–60.3	5–23.6	6–24	1–7	40–85	10–48	≤1.5
Shea (*Butyrospermum pparkii*) butter (Akihisa et al., 2010; Fontanel, 2013; Janeš and Kočevar Glavač, 2018)	—	—	3–5	30–45	—	40–60	—	4–16	<0.2	42	49	5	3–10
Argan (*Argania spinosa*) oil (Fontanel, 2013; Boucetta et al., 2014; Janeš and Kočevar Glavač, 2018)	—	—	14	6	—	45	—	35	—	20	45	35	0.7–1
Neem (*Azadirachta indica*) oil (Momchilova et al., 2007)	—	—	17.6	16.1	—	40.1	—	21.3	0.8	34	40	22	1.0–1.4
Evening Primrose (*Oenothera biennis*) oil (Ghasemnezhad and Honermeier, 2007; Fontanel, 2013; Said-Al Ahl and Ramadan, 2017)	—	—	6.2–6.7	3	—	8–13	—	70–74	<2 *γ-linolenic acid 7–10	10	9	81	1.5–2
Sacha Inchi (*Plukenetia volubilis*) oil (Soimee et al., 2020)	—	—	4.2	3.4	—	10.2	-	39.5	42.3	8	10	82	0.5
Safflower (*Carthamus tinctorius*) oil (Gürbüz and Kiralan, 2007; Matthaus et al., 2015)	<0.1	<0.2	5.6–7	1.9–2.4	<0.2	10–36.6	-	54–82	<0.1	7–10	10–40	55–83	0.6–1.5
Kukui (*Aleurites moluccana*) oil (Brown et al., 2005; Aswati and Suprihastuti, 2010; Siddique et al., 2011; Fontanel, 2013)	—	—	0–1.2	6–10	—	15–48	-	40–51	2–30	10	15–48	40–80	0.3–0.4
Borage (*Borago officinalis*) oil (Lodén and Andersson, 1996; Eskin, 2008; Fontanel, 2013; Janeš and Kočevar Glavač, 2018)	—	—	10–11	3–4	—	15	3	37–41	23–25	15	25	60	1.2–1.9
Soybean (*Glycine max*) oil (Chowdhury et al., 1970; Gunstone, 1996; Maestri et al., 1998; Bonina et al., 2005; Uzun et al., 2007; Fontanel, 2013)	—	—	10–15	3–5	—	10.6–27.5	-	50–57	2–15.6	15–19	21–25	55–60	0.5–1.7
Sunflower (*Helianthus annuus*) oil (Chowdhury et al., 1970; Fontanel, 2013; Sugawara and Nikaido, 2014; Janeš and Kočevar Glavač, 2018)	—	—	6	4	—	30	—	55	2	10	31	57	0.6–1.5

*Note: The content of individual components is based on the results of different scientific sources listed in the reference section. It is reasonable to expect that results vary slightly from study to study, as environmental factors have a significant effect on plant metabolism and consequently on the fatty acid composition. Therefore, the sum of percentages of the SFA, MUFA and PUFA content is not always 100%.

**TABLE 2:** Dermal activities of selected fatty acids; 1 = functioning of the isolated fatty acid, 2 = functioning of the isolated fatty acid in a dermal formulation, - not available.

Fatty acid	Functioning
Lauric acid (C12:0) (Anzaku et al., 2017; Janeš and Kočevar Glavač, 2018; Matsue et al., 2019; Watanabe et al., 2019)	Antimicrobial^1^
Myristic acid (C14:0) (Liu and Huang, 2012; Chen et al., 2019; Watanabe et al., 2019)	Antimicrobial^1^
Palmitic acid (C16:0) (Nguyen et al., 2015)	Antimicrobial^1^
Stearic acid (C18:0) (Khalil et al., 2000; Baumann, 2013)	Antiviral^2^
	Anti-inflammatory^2^
Palmitoleic acid (C16:1, ω-7) (Wille and Kydonieus, 2003; Janeš and Kočevar Glavač, 2018; Watanabe et al., 2019)	Antimicrobial^1^
	Penetration enhancer^2^
Oleic acid (C18:1, ω-9) (Tanojo et al., 1998; Nanayakkara et al., 2005; Mack Correa et al., 2014; Janeš and Kočevar Glavač, 2018; Watanabe et al., 2019)	Antimicrobial^1^
	Regenerative^1^
	Penetration enhancer^1,2^
Erucic acid (C22:1, ω-9)	^—^
Linoleic acid (C18:2, ω-6) (Korting and Sterry, 2001; Eichenfield et al., 2009; Zielińska and Nowak, 2014; Akinshina et al., 2016; Verallo-Rowell et al., 2016; Janeš and Kočevar Glavač, 2018)	Anti-inflammatory^1^
	Regenerative^1^
α-Linolenic acid (C18:3, ω-3) (Zielińska and Nowak, 2014; Janeš and Kočevar Glavač, 2018)	Regenerative^1^

In the context of dermal activity, the composition of vegetable butters and oils is intrinsically linked to the composition of skin lipids. Fatty acids in the skin are found in the stratum corneum (free and as structural units of ceramides) (Gray and Yardley, 1975; Hansen and Jensen, 1985; Wertz, 1992; Pappas, 2009), and in sebum (free and in diglycerides and triglycerides) (Pappas, 2009; Cunha et al., 2018). Free fatty acids in the stratum corneum are mostly saturated, with chain lengths of up to 36 carbon atoms, with tetracosanoic acid (lignoceric; C24) and hexacosanoic (ceric acid or ceratinic acid; C26) acids being the most abundant (39 M % and 23 M %, respectively) (Norlén et al., 1998). The proportion of total monounsaturated free fatty acids is approx. 20% (Van Smeden et al., 2014). Oleic (C18:1) and linoleic (C18:2) acids account for 6 and 2%, respectively, and are the only unsaturated fatty acids detected unbound in the stratum corneum (Menon et al., 2012). The human sebum is composed of triglycerides (41%), waxes (25%), free fatty acids (16%), squalene (12%), cholesterol and cholesterol esters (4%), and vitamin E (Cunha et al., 2018). Fatty acid chains range from C7 to C22 carbon atoms in length, with palmitic acid (C16) being the most abundant (Weitkamp et al. 1947; Wertz 2018). Monounsaturated fatty acids are of C14 to C18 atoms in length, the predominant acid being sapienic acid (C16:1Δ6) (Wertz 2018).

The main non-specific dermal activity of vegetable butters and oils is the emolliency of triglycerides, which results in improved skin hydration due to decreased transepidermal water loss (TEWL) (Danby et al., 2013). Specific effects include antimicrobial (Darmstadt et al., 2005; Verallo-Rowell et al., 2008), anti-inflammatory (Lucas et al., 2011) and antioxidative (Bardaa et al., 2016) action, expressed by free fatty acids and compounds of unsaponifiable matter (Poljšak et al., 2019). Dermally applied free fatty acids have also been shown to penetrate into the stratum corneum and enhance the penetration of other substances (Nanayakkara et al., 2005). Vegetable butters and oils can therefore be used to improve skin wound healing (Alves et al., 2019; Poljšak et al., 2019), ameliorate the severity of dermatitis (Desai, 2017; Hou et al., 2017), alleviate symptoms of inflammatory conditions (Styrczewska et al., 2019), etc.

The main fatty acids that express important dermal functions are briefly discussed below.

### Oleic Acid

Oleic acid is a C18:1 unsaturated ω-9 fatty acid, generally present in the majority of vegetable butters and oils. It acts as a skin penetration enhancer, as it induces permeability defects in the stratum corneum structure (Jiang et al., 2000; Mack Correa et al., 2014). The disruption of the skin’s barrier function results in an increase in TEWL (Tanojo et al., 1998; Mack Correa et al., 2014) and irritation (Tanojo et al., 1998).

### Linoleic Acid

Linoleic acid is an essential C18:2 unsaturated ω-6 fatty acid. It is a structural unit of phospholipid cell membranes, as well as ceramides in the stratum corneum, and is involved in the regulation of TEWL and lipid barrier homeostasis (Rabionet et al., 2013).

### α-Linolenic Acid and γ-Linolenic Acid

The other essential fatty acid is α-linolenic acid, a C18:3 unsaturated ω-3 fatty acid, while γ-linolenic acid is an ω-6 fatty acid. α- and γ-linolenic acids are not structural components of the skin. However, together with linoleic acid they are involved in the skin’s metabolism of polyunsaturated fatty acids (Ziboh et al., 2000). A dietary deficiency of linoleic acid, but not of α-linolenic acid, has been shown to result in skin dysfunctions such as dryness and inflammation (Ziboh and Miller, 1990).

### Unsaponifiable Compounds

While the triglyceride part of vegetable butters and oils has been researched extensively, significantly less studies have been performed on unsaponifiable compounds. This was the focus of a recently published review article from 2021 (Poljšak and Kočevar Glavač, 2021). Isolated unsaponifiable compounds were found to demonstrate wound healing, anti-acne and anti-dermatitis activities, as well as regenerative, hydrating, photoprotective and anti-wrinkle activities. However, dermal effects of unsaponifiable compounds as integral structural components of vegetable butters and oils remain largely unexplored in clinical studies. Selected unsaponifiable compounds and their dermal effects are summarized in Table 3.

**TABLE 3:** Dermal activities of selected unsaponifiable compounds; adopted from (Poljšak and Kočevar Glavač, 2021).

Unsaponifiable compound	Functioning
Phytol	Cytotoxic, autophagy- and apoptosis-inducing, anti-inflammatory, immune-modulating, antioxidative, antimicrobial
Squalene	Antitumor, anti-inflammatory, wound healing, antioxidative
Triterpene alcohols	Antitumor, anti-inflammatory, antibacterial
Phytosterols	Anti-inflammatory, antitumor, angiogenic, wound healing, antioxidative
Carotenoids	Antitumor, anti-inflammatory, antioxidative
Tocopherols and tocotrienols	Antioxidative
Flavonoids	Anti-inflammatory, antimicrobial, antioxidative
Ferulic acid	Antimelanogenesis, antioxidative, wound healing
Waxes	Anti-inflammatory, antibacterial, antioxidative
Gamma oryzanol	Anti-inflammatory, antioxidative
Phospholipids	Wound healing

## Clinical Studies

Essential progress in the understanding of the structure and functioning of the skin has been made since the first studies, which date back to about 1960 (Reinertson and Wheatley, 1959; Rawlings et al., 1994; Harding, 2004). However, in-depth investigations regarding physiological processes and the effects of individual components of the skin lipid matrix at the molecular level have only begun to emerge during the last decade (Akinshina et al., 2016; Badhe et al., 2019).

In terms of vegetable butters and oils, there is limited knowledge about their fate after dermal application, such as the extent of enzymatic hydrolysis or chemical degradation to glycerol and individual fatty acids and/or mono- or diglycerides, about penetration into the stratum corneum, inclusion in skin structures and processes, and the influence on the skin’s microbiota.

A number of *in vitro, ex vivo, in silico* and mathematical models have been developed for studying and predicting skin penetration and permeation (Moser et al., 2001; Netzlaff et al., 2007). However, none of these methodologies can thoroughly simulate real-life conditions in the human skin (Herkenne et al., 2008). Current research methods typically applied *in vivo* studies are suction blister fluid, microdialysis, skin biopsy and tape stripping. They exhibit disadvantages such as invasiveness and a lack of standardization (Herkenne et al., 2008). Among non-invasive *in vivo* methods, confocal Raman microspectroscopy is used most frequently (Darlenski et al., 2009). In general, the quantification of parameters, such as TEWL, stratum corneum hydration and skin surface acidity (pH), is essential for the integral evaluation of the lipid barrier status.

Table 4 represents a systematic review of clinical studies evaluating the effectiveness of vegetable butters and oils for dermal use. The studies were grouped according to skin condition (non-affected adult skin, infant skin, psoriasis, xerosis, UV-induced erythema, atopic dermatitis, *molluscum contagiosum*, tungiasis, and striae and scars), and listed chronologically together with the main characteristics and results.

**TABLE 4:** Clinical studies evaluating the dermal effects of vegetable butters and oils. AD—atopic dermatitis, GC—gas chromatography, MC—*molluscum contagiosum*, SA—*Staphylococcus aureus*, SC—stratum corneum, SG—striae gravidarum, SLS—sodium lauryl sulphate.

Clinical study	Population	Aim/type of the study	Application and length of the study	Oil and control	Methods	Results
Non-Affected Adult Skin						
1995 Lodén and Andersson, (1996)	21 subjects 22–57 years	Irritation Hydration	50 µL pipetted into aluminium chambers attached to the volar forearm 48 h	Borage oil Sunflower oil Canola oil Shea butter Canola oil unsaponifiables Shea butter unsaponifiables Hydrocortisone Petrolatum Fish oil Control: water	Visual evaluation of irritation Superficial blood flow (laser Doppler flowmeter) TEWL	No significant differences on non-affected skin. Significantly less visible signs of SLS-induced irritation after treatment with canola oil unsaponifiables than after treatment with water. This fraction and hydrocortisone significantly reduced blood flow. Hydrocortisone, canola oil and canola oil unsaponifiables significantly lowered TEWL.
2008 Stamatas et al. (2008)	9 subjects 30–60 years	Skin penetration Occlusion	20 µL on an area of 2 × 2 cm on the volar forearm Evaluation before application, and after 30 and 90 min	Paraffin oil Jojoba wax Almond oil Control: petrolatum	*In vivo* confocal Raman microspectroscopy Skin occlusion was assessed from the amount of SC swelling measured from the water concentration profiles	No statistical difference between paraffin oil and vegetable lipids in the extent of skin penetration and skin occlusion. Vegetable lipids demonstrated modest SC swelling (10–20%) compared to moderate swelling (40–60%) for petrolatum.
	7 infants 6–10 months		20 µL on an area of 2 × 2 cm on the volar forearm Evaluation before application and after 30 min	Paraffin oil Almond oil Control:/		
2011 Patzelt et al. (2012)	6 subjects 25–50 years	Skin penetration Occlusion	2 mg/cm^2^ of a curcumin-labelled lipid to an area of 16 cm^2^ on the volar forearm Evaluation before application and after 30 min	Jojoba wax Soybean oil Avocado oil Almond oil Paraffin oil Control: petrolatum	Laser scanning microscopy TEWL	Vegetable lipids penetrated into the first upper layers of the SC. TEWL showed that the application of the oils leads to a semi-occlusion of the skin surface; the most effective occlusion was found for petrolatum.
2015 Komane et al. (2015)	20 females 18–65 years	Irritation Single blind, controlled	On the volar forearm for 96 h	Marula oil Positive control: 1% SLS Negative control: water	Visual evaluation Surface colour determination Evaluation at 0, 24, 48, 72 and 96 h	No irritation. A statistically significant difference was observed with liquid paraffin, vaseline lotion and vaseline petroleum jelly, while marula oil resulted in marginal skin recovery. Marula oil prevented TEWL significantly, while liquid paraffin, vaseline petroleum jelly and vaseline lotion showed significantly better effects than marula oil. The occlusive effect was significant for vaseline petroleum jelly and liquid paraffin, and non-significant for marula oil and vaseline lotion. Marula oil performed better as compared to the untreated skin.
		Hydration	On the legs (calf area) for 12 days	Marula oil Controls: untreated skin; liquid paraffin, vaseline petroleum jelly, vaseline lotion	Visual evaluation Capacitance TEWL Evaluation on days 1, 2, 3, 4, 5, 8, 10 and 12	
		Occlusion	0.1 mL to an area 20 mm in a diameter on the volar forearm		Capacitance TEWL Evaluation after 0 and 30 min	
2015 Boucetta et al. (2014)	60 postmenopausal women	Hydration Randomised, open-label	Every night about 240 mg (2 mg/cm^2^) of argan oil, corresponding to 10 drops, on the left volar forearm for 60 days	Oral: argan oil Oral control: olive oil Both groups dermally: argan oil Control: untreated skin	Capacitance TEWL Evaluation on days 0, 30 and 60	The consumption of argan oil led to a significant decrease in TEWL and a significant increase in SC water content. The application of argan oil led to a significant decrease in TEWL and a significant increase in SC water content.
2019 Soimee et al. (2020)	13 females 20–60 years	Hydration Randomised, double-blind, controlled	0.5 ml on the left or right lower leg, twice a day for 14 days, followed by application discontinuation for 2 days	Sacha inchi oil Control: olive oil	Visual evaluation Capacitance TEWL Evaluation on days 0, 7, 14 and 16	Visual dryness improved, SC corneum water content improved significantly for both oils, while TEWL decreased but not significantly. The hydration capacity of both oils was equivalent. Improvement in moisture content and skin dryness for sacha inchi oil and olive oil was comparable.
Infant Skin						
2004 Darmstadt et al. (2004)	51 infants <72 h <34 weeks gestational age	Skin condition Rate of nosocomial infections Mortality Randomised, controlled	3 times daily for the first 14 days, then twice daily until 28 days of life or until discharge from the hospital Dosing: 4 g of oil per kg of body weight per treatment	Sunflower oil Control: standard skin care (i.e. minimum to no use of dermal emollients)	Visual evaluation Diagnosis of nosocomial infection	Skin condition worsened faster in the control group. Incidence of nosocomial infections was significantly reduced (54%) in the treated group. Death due to sepsis beyond the first 2 days of life was not significantly different in both groups. No side effects.
2005 Darmstadt et al. (2005)	497 infants <72 h <33 weeks gestational age	Rate of nosocomial infections Randomised, controlled	To the entire body surface, except the scalp and face, 3 times daily for the first 14 days, then twice daily until discharge Dosing: 4 g of the lipid per kg of body weight per treatment	Sunflower oil (*n* = 159) Aquaphor ointment (petrolatum, mineral oil, mineral wax, lanolin alcohol) (*n* = 157) Control: untreated (*n* = 181)	Diagnosis of nosocomial infection	Infants treated with sunflower oil were 41% less likely to develop nosocomial infections. The control lipid did not significantly reduce the risk of infections. No side effects.
2005 Solanki et al. (2005)	120 infants Gestational ages: <34 weeks, 34–37 weeks, >37 weeks	Transdermal absorption Randomised, controlled	5 ml, massaged on all available surfaces for 10 min 4 times a day, for 5 days	Safflower oil (*n* = 40) Virgin coconut oil (*n* = 40) Control: untreated (*n* = 40)	Visual evaluation Pre and post oil massage samples of blood were analysed for triglycerides and fatty acid profiles using GC.	Infants treated with safflower oil had significantly higher triglyceride and linolenic acid levels, while in the coconut oil group triglyceride and saturated fatty acid levels were significantly higher. No serious adverse events were reported; 3 infants (safflower oil group) developed a transient rash over the abdomen, which then disappeared in spite of continuing the massage.
2014 Kanti et al. (2014)	22 infants ≤48 h <37 weeks gestational age	Skin barrier development Randomised, controlled	On the whole body surface every 3–4 h during the first 10 days of life, followed by a cessation until day 21	Refined sunflower oil Control: untreated	TEWL Capacitance pH Sebum levels Microbial colonisation	In the oil group, TEWL remained stable in the forehead, while it increased significantly on the abdomen, leg and buttock skin, where—after cessation of oil application - it decreased to values comparable to the control, or in the case of the abdomen significantly below the control. No significant differences in pH, sebum levels and microbial colonisation were determined. No side effects.
2015 Nangia et al. (2015)	74 preterm infants 12 ± 6 h	Hydration Randomised, controlled	4 mL on skin of the trunk below neck, in four strokes without giving a massage, twice a day, for the first 7 days of life	Virgin coconut oil (*n* = 37) Control: standard care (*n* = 37)	Visual evaluation TEWL Microbial colonisation	TEWL was significantly lower in the oil group at all measurement points from 12 to 168 h of life. Skin condition and microbial colonisation were significantly better in the oil group.
2016 Cooke et al. (2016)	115 infants ≤72 h	The impact of dermal use on the development of atopic eczema Randomised, controlled, assessor-blinded	4 drops to the left forearm, left thigh and abdomen, twice a day for 4 weeks ± 5 days	Olive oil Sunflower oil Control: untreated	ATR-FTIR spectroscopy (structure of the SC lipid lamellae) TEWL Capacitance pH Visual evaluation	Skin condition improved in all infants, while there were no significant differences for skin surface pH and erythema scores. Hydration was significantly higher in the oil groups and the increase in the lipid ordering was significantly lower in the oil groups compared to the control.
2018 Strunk et al. (2018)	72 infants <30 weeks gestational age	Effectiveness, safety, feasibility Open-label, randomised controlled	5 mL/kg every 12 h for 21 days, starting within 24 h of birth, to the entire skin, excluding the face, scalp, and sites of catheters/drains, without massage	Virgin coconut oil (*n* = 36) Control: routine care (*n* = 36)	Visual evaluation on days 1, 7, 14, and 21	Twice-daily oil application was highly feasible, without adverse effects. Skin condition was stable in the oil group, but deteriorated in the control group.
2019 Summers et al. (2019)	995 infants ≤48 h	Skin integrity Randomised controlled	Daily full body massage for 21 days	Sunflower oil (*n* = 495) Control: mustard oil (*n* = 500)	Visual evaluation TEWL pH SC cohesion (determined as protein concentration) Evaluation on days 1, 3, 7, 10, 14, 21 and 28	Skin pH decreased faster in the sunflower oil group in the first week of life. SC cohesion was significantly higher in the sunflower oil group. Erythema, rash and dryness increased over days 1–14, then decreased by day 28, with no significant differences. TEWL increased over time, with no significant differences.
2019 Konar et al. (2020)	2,294 preterm infants <37 weeks gestational age	Effectiveness for skin maturity, prevention of sepsis, hypothermia, apnea and neurodevelopment, and safety Randomised, controlled	5 mL 4 times daily, full body massage, excluding face and scalp	Virgin coconut oil (*n* = 1,146) Control: body massage (*n* = 1,148)	Visual evaluation on days 7, 14, 21 and 28 Serum vitamin D3 on day 30 Neurodevelopment on months 3, 6 and 12 Sepsis	Significantly better skin and neurodevelopmental condition, higher weight gain and vitamin D3 level, and less hypothermia and apnea in the oil group. No significant difference in the incidence of sepsis. No significant adverse events related to the use of oil.
Psoriasis						
2005 Brown et al. (2005)	24 patients 18–78 years	Effectiveness and safety Randomised, double-blind, controlled, pilot	3 times daily, to the targeted psoriasis plaque and psoriatic lesions over the entire body, for 12 weeks	Kukui oil (*n* = 13) Control: mineral oil (*n* = 11)	Visual evaluation on weeks 0, 2, 4, 6, 8, 10 and 12	Skin condition in both groups improved, but with no significant differences between the groups. No side effects.
Xerosis						
2004 Agero and Verallo-Rowell, (2004)	34 patients 16–70 years	Effectiveness and safety Randomised, double-blind, controlled	Twice a day, on the legs, for 2 weeks	Virgin coconut oil Control: mineral oil	Visual evaluation Capacitance Sebum levels TEWL pH Evaluation on days 0, 7 and 14	Skin condition in both groups improved. SC hydration and sebum level improved significantly for both the oil and control groups, while no significant differences were found for TEWL and pH. No irritation of allergenic reaction was observed.
UVB-Induced Erythema						
2005 Bonina et al. (2005)	6 subjects 31 ± 9 years	Protection against UVB-induced erythema	200 µL immediately after exposure to UVB irradiation, on the ventral forearm	Soybean oil Control: untreated, tocopheryl acetate	Induction of erythema with an irradiation dose of twice the minimum erythema-inducing dose Evaluation after 3 h	Significant protective effect in comparison to the untreaded control and tocopheryl acetate. No side effects.
Atopic Dermatitis (AD)						
2008 Verallo-Rowell et al. (2008)	52 patients 18–40 years	Hydrating effectiveness and anti-*Staphylococcus aureus* (SA) activity Randomised, double-blind, controlled	5 mL twice a day, at two non-infected sites, for 4 weeks	Virgin coconut oil (*n* = 26) Virgin olive oil (*n* = 26)	Visual evaluation SA cultures	Significant SA antibacterial action for coconut oil. Significant improvement in skin condition for both oils, with greater effects of coconut oil.
2013 Danby et al. (2013)	Cohort 1 7 patients (self-reported AD; no symptoms for 6 months) 46 ± 5.7 years Cohort 2 12 subjects, 6 (37 ± 6.7 years) with no history of AD and 6 (32 ± 5.4 years) with a self-reported AD (no symptoms for 6 months)	Effects on adult skin barrier and implications for neonatal skin care Randomised, observer-blind, controlled	Cohort 1: 6 drops of olive oil, twice daily, on one forearm, for 5 weeks Cohort 2: 6 drops of olive oil to one forearm and 6 drops of sunflower oil to the other, twice daily, for 4 weeks	Olive oil Sunflower oil Control: untreated	pH Capacitance Erythema TEWL SC cohesion (determined as protein concentration) SC thickness	Significant increase in TEWL and decrease in SC thickness for olive oil than untreated control. Significant increase in TEWL, decrease in SC cohesion and erythema for olive oil compared to sunflower oil, in patients with a history of AD. Improved hydration and no erythema with sunflower oil.
2014 Evangelista et al. (2014)	117 patients 1–13 years	Effectiveness and safety Randomised, double-blind, controlled	5 mL, twice daily (after bath and at night), to all body surfaces (except diaper/inguinal area and the scalp), for 8 weeks	Virgin coconut oil (*n* = 59) Control: mineral oil (*n* = 58)	Visual evaluation TEWL Capacitance Evaluation on weeks 0, 2, 4 and 8	Skin condition improved in both groups, but was significantly better in coconut oil than in mineral oil group. TEWL decreased in both groups, with a significantly higher result for coconut oil than mineral oil. Skin hydration improved in both groups and was higher for coconut oil, but a significant difference only seen after 8 weeks. No side effects.
*Molluscum Contagiousum* (MC)						
2017 Kwon et al. (2017)	41 children 2–10 years	Effectiveness and safety Open	Twice daily to all lesions, for 3 months	Evening primrose oil Control:/	Visual evaluation every month	Complete or partial resolution of lesions observed in 53.7% patients. No serious adverse events related to the use of the oil, but mild inflammation was observed.
Tungiasis						
2019 Elson et al. (2019)	93 children 6–14 years	Effectiveness and safety Randomised, controlled	1 drop of oil mixture per a feet flea, on days 1 and 3 following community practice. Control: On day 1, the feet were placed up to the ankles in a basin with 2.5 L of 0.05% KMnO4 for 15 min followed by the application of petroleum jelly over the whole foot	20% virgin neem oil and 80% virgin coconut oil (*n* = 48) Control: 0.05% KMnO4 foot bath (*n* = 45)	Evaluation of pain and itching *Tunga penetrans* viability Evaluation on days 0, 1, 3, 5 and 7	Significant, but similar flea mortality was observed for both groups. Faster aging of fleas and less pain in the oil group, but with no significance. No side effects.
Striae gravidarum (SG), striae and scars						
2011 Taavoni et al. (2011)	70 pregnant women 20–30 years 18–20 weeks gestational age	Effectiveness in striae gravidarum (SG) Randomised	Twice daily on the abdominal area without massaging, for 8 weeks	Olive oil (*n* = 35) Control: untreated (*n* = 35)	Occurrence of SG Weekly evaluation	Among women without previous striae, SG occurred in 54.3% women using olive oil and 37.1% control women. Among women with striae present, SG occurred in 45.7% women using olive oil and 62.9% control women. However, there was no significant difference.
2012 Soltanipour et al. (2012)	100 pregnant women 20–30 years 18–20 weeks gestational age150 pregnant women	Effectiveness in SG Randomised, controlled	1 cm^3^ twice daily on the abdominal skin, without massaging, until delivery	Olive oil (*n* = 50) Control: untreated (*n* = 50)	Occurrence of SG Evaluation on 37–40 weeks gestational age	The incidence and severity of SG were lower in the olive oil, but with no significant difference.
2014 Soltanipour et al. (2014)	20–30 years 18–20 weeks gestational age	Effectiveness in SG Randomised, assessor-blinded, controlled	1 cm^3^ twice daily on the abdominal skin, without massaging, until delivery	Olive oil (*n* = 50) Saj^®^ cream (*n* = 50) Control: untreated (*n* = 50)	Occurrence of SG Evaluation on 38–40 weeks gestational age	No significant differences among the three studied groups regarding the incidence and severity of SG.
2017 Bielfeldt et al. (2018)	80 subjects 35.3 ± 12.0 years	Effectiveness in scars and striae, and safety Randomised, assessor-blinded, controlled	Twice daily, for 8 weeks	Safflower oil (55.9%), olive oil (42%), grapefruit (Citrus grandis) peel essential oil (2%) oil, tocopherol (0.1%) Control: untreated	Visual evaluation on day 57	Skin condition improved significantly. No side effects.

## Discussion

The dermal use of vegetable butters and oils probably dates back to the times of Ancient Egypt. Today, scores of different butters and oils are available for therapeutic and cosmetic purposes, and are researched in scientific studies. While their chemical composition has been studied in detail, significantly less research has been done to elucidate the mechanisms of action after application on the skin, particularly at the level of clinical effectiveness in the treatment of skin disorders (Janeš and Kočevar Glavač, 2018). Surprisingly, systematic studies were not available until the 1990s (Lodén and Andersson, 1996). Research then intensified after 2010 and, in the last few years, increased interest is reflected in comprehensive review articles (Lin et al., 2017; Vaughn et al., 2018; Poljšak, Kreft, and Kočevar Glavač, 2019; Moore, Wagner, and Komarnytsky, 2020). The reasons for the latter primarily derive from direct evidence that vegetable butters and oils function as effective active pharmaceutical ingredients in dermal treatments, and as cosmetically active ingredients in cosmetics, as evident from the clinical studies reviewed in Table 4. They are also generally linked to good skin compatibility, have fewer side effects, are affordable and easily accessible. Finally, in terms of today’s patients/consumers, we cannot neglect the fact that they are being increasingly used as alternatives for synthetic actives simply due to their natural origin.

The reviewed clinical studies on non-affected skin (Table 4) focused mainly on investigating penetration capacity, occlusive/hydrating effects and irritation potential, and included argan (*Argania spinosa*) oil, borage (*Borago officinalis*) oil, rapeseed (*Brassica napus*) oil, shea (*Butyrospermum parkii*) butter, soybean (*Glycine max*) oil, sunflower (*Helianthus annuus*) oil, olive (*Olea europaea*) oil, avocado (*Persea americana*) oil, sacha inchi (*Plukenetia volubilis*) oil, almond (*Prunus dulcis*) oil and marula (*Sclerocarya birrea*) oil (Lodén and Andersson, 1996; Stamatas et al., 2008; Patzelt et al., 2012; Boucetta et al., 2014; Komane et al., 2015; Soimee et al., 2020). The oils were proven to be semi-occlusive, which resulted in decreased levels of TEWL and/or increased stratum corneum hydration. Hydration was shown to improve very soon after application (30 min) and lasted for the duration of the studies (from 1 day to 60 days). The occlusive effects on non-affected skin were comparable to those of the controls (usually petrolatum or mineral oil), and were also directly confirmed in a clinical study with coconut (*Cocos nucifera*) oil on xerotic skin (Agero and Verallo-Rowell, 2004).

In this review, we placed special emphasis on the evaluation of possible connections between the fatty acid composition of triglycerides and the negative effects of the oils on the stratum corneum structural integrity. The skin’s lipid barrier disruption is assumed to be connected to vegetable oils with a content of predominantly oleic acid in triglycerides, and it was suggested that the ratio of oleic acid to linoleic acid may be crucial (Vaughn et al., 2018; Poljšak, Kreft, and Kočevar Glavač, 2019). However, in the case of non-affected skin, the reviewed vegetable oils were proven to be non-irritating, and this seems to be independent of the fatty acid composition. Sacha inchi and olive oils showed comparable effects and both were beneficial for dry skin (Soimee et al., 2020). Yet, their fatty acid composition is significantly different, with an approx. 1:4 ratio of oleic acid (10.2%) to linoleic acid (39.5%) for sacha inchi oil (Soimee et al., 2020), while oleic acid is typically predominant (>70%) over linoleic acid (10%; Table 1) in olive oil. Similar findings showing no irritation were reported for marula oil (69.0% oleic acid, <10% linoleic acid) (Komane et al., 2015). The resistance of the skin to the potentially damaging effects of vegetable oils with a high content of oleic acid in triglycerides may be explained by the physiological mechanisms of barrier repair in healthy skin not suffering from pathological conditions. Furthermore, almond, rapeseed and avocado oils represent vegetable oils with a 2–3:1 ratio of oleic acid to linoleic acid (Table 1), which corresponds closely to the physiological ratio of 3:1 (Menon et al., 2012), while argan oil has a ratio of approx. 1:1. However, no significant skin benefits were identified in connection with this ratio (Lodén and Andersson, 1996; Stamatas et al., 2008; Patzelt et al., 2012).

We conclude that studies have not yet proven whether the physiological ratio of oleic acid to linoleic acid could be considered a boundary between the positive and negative skin effects of dermally applied triglycerides. Moreover, non-affected skin is capable of resisting the damaging potential to disrupt the stratum corneum structure, resulting from the dermal use of vegetable oils with a high content of oleic acid in triglycerides.

Nine clinical studies (Darmstadt et al., 2004, 2005; Solanki et al., 2005; Kanti et al., 2014; Nangia et al., 2015; Cooke et al., 2016; Strunk et al., 2018; Summers et al., 2019; Konar et al., 2020) performed on infant skin explored the effects of safflower (*Carthamus tinctorius*) oil, coconut (*Cocos nucifera*) oil, sunflower (*Helianthus annuus*) oil, olive (*Olea europaea*) oil and mustard (*Sinapis alba*) oil. The results revealed a low cost, availability, simplicity, beneficial action and the effectiveness of treatments. Based on studies evaluating sunflower oil (Darmstadt et al., 2004; Darmstadt et al., 2005), vegetable oils were identified as an important intervention for treating infants in developing countries, especially for the reduction in the incidence of nosocomial infections. In addition, the oils significantly reduced TEWL and improved hydration, and generally no side effects were observed. In contrast to non-affected adult skin, the oil composition seems to be important for maintaining lipid barrier integrity in infants (Summers et al., 2019). Moreover, the skin’s structural integrity may be even more affected by the frequency and duration of the oil’s use. An increase in TEWL and a decrease in hydration was identified after the application of a refined sunflower oil every three to four hours. It is not clear, however, if low-oleic acid or mid-oleic acid sunflower oil was used in the study (Kanti et al., 2014). Oleic acid-rich triglycerides of olive oil were previously shown to damage the lipid barrier integrity in adult skin (Danby et al., 2013). Based on the aforementioned negative effect of the frequent use of refined sunflower oil every three to 4 hours (Kanti et al., 2014), the importance of unsaponifiable matter may also be taken into account. Finally, we must emphasize that in terms of long-term safety, it is advisable to use vegetable butters and oils on infants only when necessary, as the penetration of dermally applied oils through the non-mature skin of babies was found to be significant because the triglyceride profile in blood changed after an oil massage four times a day for five days (Solanki et al., 2005).

Coconut, sunflower and olive oils were used in three studies on skin affected by atopic dermatitis (Verallo-Rowell et al., 2008; Danby et al., 2013; Evangelista et al., 2014). Coconut oil, characterized by the predominant saturated fatty acids in triglycerides, was superior to mineral oil (Evangelista et al., 2014). In addition, treatment with coconut oil resulted in a significantly decreased *Staphylococcus aureus* colonization in comparison to olive oil (Verallo-Rowell et al., 2008). As expected, olive oil was proven not to be a good option for the treatment of atopic dermatitis. Oleic acid-rich triglycerides of olive oil (76.3% oleic acid, 4.6% linoleic acid) damaged the lipid barrier integrity, while sunflower oil (27.3% oleic acid, 60.9% linoleic acid) with an approx. 1:2 ratio of oleic acid to linoleic acid did not disturb the stratum corneum integrity, caused no erythema and improved skin hydration in adults with and without a history of atopic dermatitis (Danby et al., 2013).

Other skin conditions or diseases have been researched to a lesser extent. One study (Brown et al., 2005) investigated the effect of kukui (*Aleurites moluccana*) oil on psoriasis. Kukui oil was characterized by an approx. 1:2 ratio of oleic acid (21.21%) to linoleic acid (41.27%) in triglycerides. The oil had a positive effect but the reduction of symptoms was not significant compared to the effects of the control oil (mineral oil). In 2005, a study was conducted that evaluated the ability of soybean (*Glycine max*) oil to protect the skin from UVB-induced erythema (Bonina et al., 2005). Experiments showed that soybean oil (10.6% oleic acid, 56.5% linoleic acid) with an approx. 1:5 ratio of oleic acid to linoleic acid exhibited beneficial protective activity, which was stronger than that of tocopheryl acetate. A study evaluating the dermal use of evening primrose (*Oenothera biennis*) oil in children with *molluscum contagiosum* confirmed the potential for therapeutic treatment. The study, however, was not controlled (Kwon et al., 2017). For treating tungiasis, a mixture of 20% neem (*Azadirachta indic*a) oil and 80% coconut oil was compared to 0.05% KMnO_4_ (Elson et al., 2019). The antiparasitic effectiveness of the oil mixture against *Tunga penetrans* was not superior to that of KMnO_4_. However, secondary outcomes were better. In terms of the composition of this oil mixture, coconut oil contributes mainly saturated fatty acids, with the predominant acid being lauric acid, while neem oil is characterized by an approx. 1:2 ratio of oleic acid to linoleic acid. Researchers stressed that the compounds of neem oil unsaponifiable matter (azadirachtin, azadirachtin derivatives and salanin) contribute significantly to the antiparasitic activity.

In the aforementioned studies, inflammation was the main process controlling/affecting skin conditions/disorders. We conclude that in inflammation-affected skin, vegetable oils with a highly dominant content of oleic acid, together with the lack of or a low linoleic acid content, may cause additional disruptive changes to the stratum corneum structure. This may result in an increase of TEWL and a decrease in hydration, and in erythema. In contrast, beneficial dermal effects may be expected in inflammation-affected skin from vegetable oils and their mixtures with a high content of linoleic acid in triglycerides.

Vegetable butters and oils are also frequently used in the prevention and treatment of striae. Three studies (Taavoni et al., 2011; Soltanipour et al., 2012; Soltanipour et al., 2014) investigating olive oil were conducted, but no significant effects were observed in reducing the incidence and severity of striae. However, positive results were reported for a body oil composed of 55.9% safflower oil, 42% olive oil, 2% grapefruit (*Citrus grandis*) essential oil and 0.1% tocopherol (Bielfeldt et al., 2018). The fatty acid composition of triglycerides supports a beneficial contribution of linoleic acid to the overall effect.

Finally, although the effectiveness of vegetable butters and oils for the improvement of skin conditions, or prevention and treatment of skin diseases is supported by clinical evidence, some of the conclusions that we have drawn must be further studied and backed up by new research of high quality. Namely, limitations of the reviewed clinical studies generally include a small number of patients, heterogeneity in terms of study design and duration, methods of evaluation, dosing regimen, and an unknown composition of the fatty acid profile and unsaponifiable compounds.

## Conclusion

The reviewed studies focused on the effects of 17 vegetable oils on non-affected skin, infant skin, psoriasis, xerosis, UVB-induced erythema, atopic dermatitis, *molluscum contagiosum*, tungiasis, scars, striae and striae gravidarum. Coconut, olive and sunflower oils appeared most frequently, which demonstrates their availability and recognition in terms of a long history of dermal use. However, less-known and newly discovered oils are gaining attention.

The reviewed clinical studies show the importance of vegetable butters and oils as therapeutically and cosmetically active ingredients for dermal use. Chemical composition of both the triglyceride fraction and unsaponifiable matter is the basis for the comprehensive understanding of mechanisms of action and effects after their application on the skin, and enables a customized approach for the treatment of skin diseases and cosmetic care of the skin. However, a lack of knowledge of how vegetable butters and oils and their components are metabolized and/or incorporated in the skin following dermal application, and how they affect the structure and properties of the lipid matrix as well as the skin’s microbiota call for further research.
